# The PREMISE database of 20 *Macaca fascicularis* PET/MRI brain images available for research

**DOI:** 10.1038/s41684-023-01289-9

**Published:** 2023-11-23

**Authors:** Lucie Chalet, Justine Debatisse, Oceane Wateau, Timothe Boutelier, Marlène Wiart, Nicolas Costes, Inés Mérida, Jérôme Redouté, Jean-Baptiste Langlois, Sophie Lancelot, Christelle Léon, Tae-Hee Cho, Laura Mechtouff, Omer Faruk Eker, Norbert Nighoghossian, Emmanuelle Canet-Soulas, Guillaume Becker

**Affiliations:** 1 CarMeN Laboratory, Université Claude Bernard Lyon 1, INSERM U1060, INRA U1397, Lyon, France; 2 Olea Medical, La Ciotat, France; 3 Institut des Sciences Cognitives Marc Jeannerod (ISCMJ), UMR 5229 CNRS, Bron Cedex, France; 4 Cynbiose SAS, Marcy-L’Etoile, France; 5 CERMEP, Lyon, France; 6 Hospices Civils de Lyon, Lyon, France; 7 CREATIS, CNRS UMR 5220, INSERM U1206, Université Lyon 1, INSA Lyon, Bât. Blaise Pascal, Villeurbanne, France; 8 Present Address: Lyon Neuroscience Research Center, University Claude Bernard Lyon 1, INSERM U1028, CNRS UMR 5292, Lyon, France

**Keywords:** Neuroscience, Databases

## Abstract

Non-human primate studies are unique in translational research, especially in neurosciences where neuroimaging approaches are the preferred methods used for cross-species comparative neurosciences. In this regard, neuroimaging database development and sharing are encouraged to increase the number of subjects available to the community, while limiting the number of animals used in research. Here we present a simultaneous positron emission tomography (PET)/magnetic resonance (MR) dataset of 20 *Macaca fascicularis* images structured according to the Brain Imaging Data Structure standards. This database contains multiple MR imaging sequences (anatomical, diffusion and perfusion imaging notably), as well as PET perfusion and inflammation imaging using respectively [^15^O]H_2_O and [^11^C]PK11195 radiotracers. We describe the pipeline method to assemble baseline data from various cohorts and qualitatively assess all the data using signal-to-noise and contrast-to-noise ratios as well as the median of intensity and the pseudo-noise-equivalent-count rate (dynamic and at maximum) for PET data. Our study provides a detailed example for quality control integration in preclinical and translational PET/MR studies with the aim of increasing reproducibility. The PREMISE database is stored and available through the PRIME-DE consortium repository.

## Abstract (web summary)

The authors present a simultaneous PET/MR dataset of 20 *Macaca fascicularis* images structured according to the Brain Imaging Data Structure standards. The PREMISE database is stored and available through the PRIME-DE consortium repository.

## Main

In the framework of open science, sharing imaging databases offers specific benefits in terms of analytical tools development and validation^1^. According to the FAIR Data Principles, the development of accessible imaging databases will help increase the reproducibility between studies^2^. In this respect, neuroimaging scientists examine their respective policies and practices^3^. This is also valid in preclinical research where in vivo imaging of non-human primates (NHPs) holds great potential in comparative biology and biomedical research^4,5^. NHP neuroimaging databases enable the scaling of findings for cross-species comparative and translational neurosciences and a better understanding of brain regions’ functions in health and disease. Besides, the establishment and sharing of preclinical neuroimaging databases complies with the 3R principles, especially reduction through the use of imaging datasets, and refinement considering that in vivo imaging is non-invasive and favors clinical translation. Therefore, preclinical neuroimaging data sharing associates open science objectives and 3R principles toward better reproducibility and transparency in research^6^. However, several challenges must be overcome by the NHP neuroscience research community. Historically, single-lab imaging protocols and heavy logistics of research studies have resulted in data acquisition inconsistency and discrepancy of obtained results^7^. Ultimately, this limitation may compromise appropriate data comparison between research groups. The NHP research community is currently facing a substantial challenge due to the scarcity of animals. The worldwide sanitary crisis caused by severe acute respiratory syndrome coronavirus 2 has severely impacted the already precarious supply chain for these animals, and the drastic price increase for experimental NHP might strongly impact biomedical research^8,9^.

In recent years, the NHP research community has moved forward to tackle challenges ahead, most notably the limited availability of data. The PRIMatE Data Exchange (PRIMatE-DE) initiative addresses this challenge by aggregating independently acquired NHP in vivo imaging datasets^10^. Initially intended for magnetic resonance imaging (MRI) data, the community has worked to standardize data collection with minimal acquisition specifications, and data architecture allowing data sharing within the framework of open science^10^. This collaborative work allowed us to gradually improve our neuroimaging studies to human standards. In this context, the Brain Imaging Data Structure (BIDS) became the gold standard for organizing and sharing neuroimaging datasets^1^. Nuclear imaging specialists jumped on board and published guidelines to improve the accuracy and sharing of positron emission tomography (PET) data^11–13^. Therefore, while PET neuroimaging databases exist, their counterparts in NHP are either missing or not easily available. Furthermore, considering the development of hybrid PET/magnetic resonance (MR) scanners and their translational potential in NHP neuroimaging studies, there is a growing need for NHP PET/MR hybrid imaging databases.

In this Article, we developed a multi-modal database of *Macaca fascicularis*, acquired on a clinical PET/MR scanner, and constructed with MRI, [^15^O]H_2_O and [^11^C]PK11195 PET images. [^15^O]H_2_O is a freely diffusible PET tracer used to measure brain perfusion and considered as a reference for non-invasive cerebral blood flow quantification^14^. [^11^C]PK11195 is a PET radiotracer specifically targeting the translocator protein 18 kDa (TSPO) and is widely used to investigate brain inflammation in relation to various neurological disorders and notably brain ischemia^15,16^. The data were formatted to BIDS standards using a self-designed Python script compensating for missing metadata frequently encountered with preclinical and retrospective data. The designed script is available in open access on GitLab. The entire dataset is available upon request from the PRIMatE-DE repository.

## Results

**Fig. 1:**
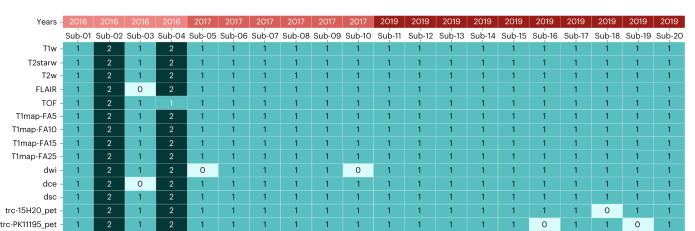
Heatmap representation of data availability in the shared BIDS database for each subject with associated acquisition year. Darker columns highlight two subjects with test–retest acquisition sessions. FA, flip angle; FLAIR, fluid-attenuated inversion recovery; T1map, T1 mapping; T1w, T2w, T2starw, relaxation weighted; TOF, time of flight; trc, tracer.

The collection of participants’ information enabled a detailed description of the cohort’s age (6 ± 0.65 years) and weight (7.37 ± 1.11 kg). A summary of the age and weight distribution is provided in Table 1. After converting the data to BIDS standards, the sequences’ availability was assessed using an automated counter. The data availability is provided in Fig. 1. This analysis highlighted the presence of a test–retest for two subjects in the dataset and a few missing acquisitions. The lack of data is explained by issues with radiosynthesis, contrasting agents or movement during acquisition for PET, contrast imaging and MRI sequences, respectively. This figure also indicates the study year for each subject, ranging from 2016 to 2019. This parameter is relevant to explain variability in data quality due to software updates on the PET–MRI system.

**Table 1:** Cohort subject’s weight and age distribution

		Population	Percentage of population
**Age (years)**	5	4	20
	6	12	60
	7	4	20
**Weight (kg)**	[5.5;7.0]	8	40
	[7.0;8.5]	8	40
	[8.5;10]	4	20

**Fig. 2:**
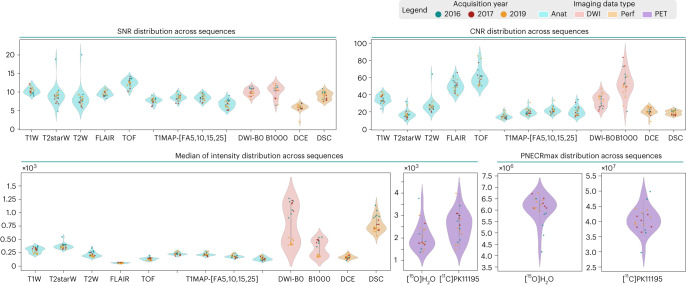
Quality of PET and MRI acquisitions included in the shared dataset. Quality is expressed in SNR, CNR, median of intensity and maximum of PNECR distributions across subjects and sequences. Anat, anatomical sequences; DWI, diffusion-weighted imaging; FA, flip angle; FLAIR, fluid-attenuated inversion recovery; Perf, perfusion sequences; PET, positron emission tomography; T1map, T1 mapping; T1w, T2w, T2starw, relaxation weighted; TOF, time of flight.

The quality of the acquisitions in the shared BIDS database is expressed with signal-to-noise ratio (SNR), contrast-to-noise ratio (CNR) and median of intensity. The quality assessment highlighted the consistency of the acquisition quality (averaged and over time) as displayed in Fig. 2.

While acquisition quality was highly reproducible, an outlier in T_2_-weighted and T_2_^*^-weighted sequences was identified. This outlier is due to a variation in acquisition parameters during session 2 of subject 4 as shown in Table 2. The dynamic contrast-enhanced (DCE)-perfusion acquisitions also uncovered an outlier due to the movement of subject 15 during acquisition.

**Table 2:** Parameters of MRI sequences

Sequences	Years	Pulse sequence	Acquisition type	Echo time	Number of echo time	Repetition time	Flip angle	Pixel bandwidth	Acquisition matrix	Pixel spacing
T1w	2016, 2017, 2019	MPRAGE	3D	0.00421	1	2.65	9	190	[256, 256]	[0.62, 0.62, 0.6]
T2starw	2016, 2017	GR	3D	0.00445, 0.0043^a^	16, 12^b^	0.205, 0.147^b^	20	219	[128, 128], [256, 256]^b^	[1.0, 1.0, 1.0]
	2019			0.00445	16		25	220	[128, 128]	
T2w	2016	SE	2D	0.0099	32	6.46, 3.23^b^	180	262	[64, 64], [128, 128]^b^	[2.0, 2.0, 2.0]
	2017					7.11			[64, 64]	
	2019							260		
FLAIR	2016, 2017	IR	3D	0.419	1	5	120	554	[192, 192]	[0.42, 0.42, 0.8]
	2019			0.418				555		
TOF	2016, 2017	GR	3D	0.00357, 0.00359^a^	1	0.0212	20	243	[448, 220]	[0.45, 0.45, 0.9]
	2019			0.00357		0.022	20	245		
T1map-FAx	2016, 2017	GR	3D	0.0025	1	0.0052	5, 10, 15, 25	401	[64, 64]	[2.0, 2.0, 2.0]
	2019							400		
dwi	2016, 2017	DWI	2D	0.068	1	11.20	180	898	[96, 96]	[1.33, 1.33, 1.3]
	2019			0.057		6.65		965		
dce	2016, 2017	GR	3D	0.0025	1	0.0052	25	401	[64, 64]	[2.0, 2.0, 2.0]
	2019							400		
dsc	2016	EPI	2D	0.017	1	1.5	90	1698	[64, 64]	[2.0, 2.0, 2.0]
	2017					1.0				
	2019					1.04		1700		

DSC contrast agent: 4 ml Dotarem (Guerbet) followed by intravenous injection of 10 ml saline (injection rate, 3 ml/s) using a power injector (MEDRAD, Bayer). DCE contrast agent: 0.1 mmol Gd/kg at 3 ml/s of Dotarem, followed by 10 ml saline flush at the same rates.

EPI, echo planar imaging; FA, flip angle; FLAIR, fluid-attenuated inversion recovery; GR, gradient echo; IR, inversion recovery; SE, spin echo; TOF, time of flight.

^a^sub-01_ses-01.

^b^sub-04_ses-02.

Two groups can be discriminated in the diffusion-weighted imaging (DWI) metrics evaluation. This distinction is due to a software update in 2019 after which the sequence’s parameters were strongly modified for this acquisition. As a result of the software update, the median intensity is considerably reduced. A variation in SNR and CNR can also be observed, but with a milder difference between software versions.

**Fig. 3:**
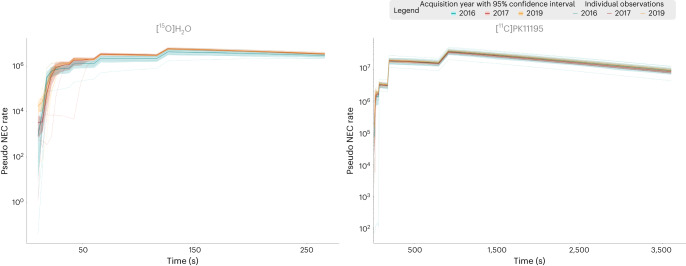
Quality of shared dataset PET scans. Quality is expressed with PNECR evolution over time expressed on logarithmic scale for both [^15^O]H_2_O and [^11^C]PK11195 radiotracers. NEC, noise-equivalent count.

A variability in the PET signal intensity can be observed for both tracers. The maximum of pseudo-noise-equivalent count rate (PNECR) metric shows a difference between 2016 acquisitions and later campaigns. These early data show a lower maximum of PNECR with [^15^O]H_2_O tracer and a wider variability across subjects with [^11^C]PK11195 tracer. Figure 3 shows the evolution of PNECR over time for both tracers expressed on a logarithmic scale. This representation highlights the variability in tracer injection time for acquisitions before 2017.

**Fig. 4:**
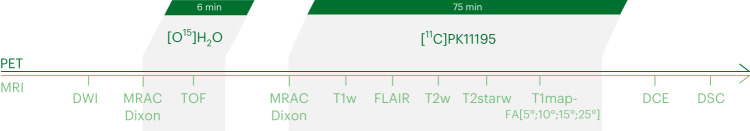
Timeline for simultaneous PET–MRI acquisition protocol. DCE, dynamic contrast enhanced; DSC, dynamic susceptibility contrast; DWI, diffusion weighted imaging; FA, flip angle; FLAIR, fluid attenuated inversion recovery; MRI, magnetic resonance imaging; PET, positron emission tomography; T1map, T1 mapping; T1w, T2w, T2starw, relaxation weighted; TOF, time of flight.

While we uncovered acquisition parameters variations throughout acquisition years, the dataset protocol used did not vary. Figure 4 shows the simultaneous acquisitions of MRI sequences and PET scans.

## Discussion

In line with human neuroimaging advances, the NHP scientific community is evolving toward more reproducibility and transparency in biomedical sciences, notably through the means of data sharing. To the best of our knowledge, this NHP imaging database is the first open-source collection including simultaneous dynamic PET-acquisition and MRI sequences. We provide a wide range of NHP MRI acquisitions for download, including structural, diffusion and perfusion imaging.

BIDS standards were originally designed to guide best practices for the storage and sharing of fMRI datasets^1^. PET imaging modalities came afterwards as extensions to the original BIDS specifications^12^. In this context, Drs. Gitte Knudsen and Robert Innis initiated a collaborative working program to address specific PET imaging challenges through the definition of standards for organizing and sharing^11^. We followed the current recommendations regarding the description of data acquisition and reconstruction methods as well as the molecular description of the radiotracers.

The purpose of the present brain NHP database is to provide the molecular imaging community with a full dataset with detailed quality descriptions. We included quality control measures of PET images using the median intensity metric. Although the methods we propose here are derived from MRI applications, we believe that they can be used to quantify the variability of the different PET data acquisitions. This variability is due to the injected radioactivity, subject’s weight and physiological constants’ variations. While corrections can be applied to normalize weight and dose variations using standardized uptake value quantification, variability of physiological constants can be compensated using reference region ratios (standardized uptake value ratios). We provided uncorrected data enabling future users to apply their own normalization and modeling methods. We assessed the variability of our PET data using the PNECRmax, which showed a high reproducibility for [^15^O]H_2_O tracer given the highly standardized injected dose; however, the data show a variability in the timing of injection for the scans acquired in 2016. The variability in PNECRmax is slightly higher for [^11^C]PK11195 radiotracer, due to a larger range of injected dose. However, [^15^O]H_2_O data displayed more noise compared with [^11^C]PK11195 data due to the shorter frame duration and the extremely short half-life of [^15^O]. We analyzed the dynamic evolution of PNECR, which was stable over time and between animals, except for early frames corresponding to the bolus entry into the brain. As expected the counting rate is almost ten times higher for the [^11^C]PK11195 than for the [^15^O]H_2_O.

We are aware of the quality limitations of the MRI data in comparison with the standards in NHP imaging^17^. These drawbacks are largely due to the specifics of our research protocol dedicated to translational stroke research. Therefore, baseline acquisitions (before stroke induction) were acquired in the same conditions as further occlusion–reperfusion acquisitions^18,19^, which precludes the use of a stereotaxic frame for a repeatable animal position in the scanner. Moreover, due to the specificity of our model, acquisitions had to be shortened to fit experimental conditions in stroke phases. While we observed variability in our data quality that might alter the automation of pipelines, the diversity of our database can represent a source of interest for a wide range of applications from noise reduction to anatomical studies. Furthermore, simultaneous acquisitions enable modality comparison; for instance our database provides perfusion imaging with MRI (dynamic susceptibility contrast, DSC) and PET ([^15^O]H_2_O) for potential cross-comparative studies of the resulting parameters.

When formatting a retrospective and/or preclinical database to BIDS standards, we frequently encounter missing data and missing Digital Imaging and Communications in Medicine (DICOM) tags. These inconsistencies are difficult to identify and compensate. Therefore, we found a need for a self-designed tool to format data following BIDS guidelines. Additionally, this tool enables the selection of specific setting/experimental phase to include in the converted database. We think this functionality could encourage scientists to share parts of their data while holding the remaining settings until results are published. The tool also supports a variety of raw data folder organization, as we know it varies between structures and institutes. A common issue faced in data formatting and sharing is the time and resources these initiatives require. This issue highlights the need for data scientists dedicated to these tasks in research teams. We are hoping that the provided tool will facilitate such initiatives that are urgently needed to address challenges raised by the use of NHP in biomedical research.

To conclude, we generated an original and diverse NHP hybrid PET/MR database available for the community through PRIME-DE platform and hope that the present work, describing the quality of the published data and metadata, will encourage the neuroimaging community to use it.

## Methods

### Animal cohort description

This dataset, which includes 20 mature male cynomolgus macaques (*M. fascicularis*), was generated using baseline images from the primate stroke model described by Debatisse et al. and Becker et al. in 2021 and 2023, respectively^16,18^. The experimental protocol was approved by the Animal Welfare Body of Cynbiose and the Ethics Committees of VetAgro-Sup and CELYNE CEEA n°42 and was carried out in accordance with the European Directive 2010/63/UE and ARRIVE guidelines (Animal Research: Reporting in Vivo Experiments)^20,21^.

The subjects underwent combined PET–MRI acquisitions following anesthesia induced by intramuscular injection of ketamine (4 mg/kg; KetamineVR 1000, Virbac) and midazolam (1.3 mg/kg; MidazolamVR 5 mg/ml, Mylan). Sevoflurane (1%, variable depending on the animal’s anesthetic depth; SevoFloVR, Abbott Laboratories) maintained anesthesia during acquisition. Animals were intubated and monitored through heart and respiratory rate, end-tidal CO_2_, systolic, diastolic and mean arterial pressure, oxygen saturation and body temperature.

### PET–MRI acquisitions

PET–MRI sequences were acquired on a fully integrated hybrid Biograph mMR PET-MRI 3T Siemens scanner (Siemens Healthcare). Imaging acquisitions were conducted between 2016 and 2019 with software versions ‘syngo MR B20P’ and ‘syngo MR E11’.

MRI sequences are previously described in Results with corresponding parameters according to years of acquisition (Table 2).

Images from PET radiotracers [^15^O]H_2_O (255 ± 15 MBq) and [^11^C]PK11195 (140.1 ± 21.4 MBq) were acquired for 6 min and 70 min, respectively, after bolus injections. While molar activity could be measured for [^11^C]PK11195 (48.0 ± 24.5 GBq/μmol) providing information on injected mass (3.50 ± 1.7 nmol), the half-life of [^15^O]H_2_O did not permit such precise radioactivity measurements. The data were reconstructed on a 256 × 256 × 127 matrix (voxel size 0.7 × 0.7 × 2.0 mm^3^), 26 cm axial field of view using a point-spread function and ordinary poisson ordered subset expectation maximization (OP-OSEM) iterative reconstruction method including normalization as well as correction for attenuation, scatter, random counts and dead time. Before the PET–MRI session, a computed tomography scan (Siemens Biograph mCT64, Siemens Healthcare) was obtained for each animal and used for PET attenuation correction. [^11^C]PK11195 dynamic PET data were reconstructed in 28 frames: 6 × 10 s, 6 × 20 s, 6 × 120s and 8 × 300s. [^15^O]H_2_O dynamic PET were reconstructed in 26 frames: 8 × 4 s, 4 × 6 s, 6 × 10 s and 8 × 20s. Lastly, a post-reconstruction 3D Gaussian filter of 4 mm was applied.

### Data processing

The data processing pipeline consisted in formatting datasets to BIDS^1^. Existing formatting tools such as dcm2bids^22^ have limited support for missing DICOM tags frequently encountered in preclinical and retrospective datasets. Therefore, an automated Python script compatible with MRI and PET acquisitions was developed. Raw images are loaded in DICOM format and converted to NIfTI format with associated metadata in JSON format in accordance with BIDS guidelines. An overview of the Python script tasks is provided in Supplementary Fig. 1. The script’s ambition is to provide flexibility in data organization and selection when formatting raw data for sharing purposes. Because raw-data folder organization varies between structures, additional parameters are provided enabling the definition of the raw folder structure. For instance, single-session and multiple-session studies are supported by the pipeline. It is also possible to select which acquisitions and sessions to format to BIDS. This option may be useful in multi-phase studies in which data exploration of key phases delays data sharing of baseline acquisitions. Additionally to the data conversion, the script provides templates for mandatory BIDS files such as dataset description and participants list.

### Converting pixel data to NIfTI image

DICOM volume loading and conversion to NIfTI format is performed using the pydicom and nibabel Python packages^23,24^. These packages were integrated in a Python class handling multi-dimensional volumes from lists of DICOM files enabling the building of pixel data arrays and managing data orientation to improve NIfTI encoding. Limitations remain for oblique orientations, frequently encountered in large animal models; special care should be provided when handling NIfTI conversions, and manual corrections might be necessary. Therefore, the orientation of the pixel data was manually validated using the ITK-snap software^25^. The provided data were acquired on a clinical PET–MRI system without a stereotaxic frame; therefore, a unique subject position was established close to a patient position. All images are shared in raw space; no registration to standard atlas space was applied. The oblique encoding of MRI sequences led to potential errors in conversions. The closest orientation was right–superior–anterior for the majority of subjects. Other subjects were oriented oblique closest to right–anterior–inferior. To provide uniform data, the identified right–anterior–inferior subject’s data were manually oriented to right–superior–anterior. To match PRIME-DE orientation homogeneity criteria, the full dataset was then oriented to right–posterior–inferior. Additionally, DWI and diffusion tensor imaging require bval and bvec files to qualify the sequences. These files are obtained with the dicom2nifti Python package^26^.

### Generating the metadata file

To collect the necessary metadata for each acquisition sequence, compensate missing tags and integrate subject-specific radioactivity parameters, three configuration files are necessary.

#### Sequence overview

The sequence overview configuration file provides a list of sequences to integrate in the BIDS formatting. It provides details on tags necessary for each sequence and replacement values if the tag is missing in the DICOM metadata. In this configuration file, the naming format for the sequence in the BIDS database is also defined along with instructions on where to find and store the corresponding data. Supplementary Table 1 provides a description of each column required in the sequence overview configuration file along with examples.

#### PET doses

The PET-specific configuration file enables the definition of each subject’s injected radioactivity parameters. The file sets values for BIDS-guideline required PET tags SpecificRadioactivity (that is, molar activity, the current term recommended by the guidelines^27^), InjectedRadioactivity and InjectedMass. This file is required only for PET data formatting. Configuration file description and examples are provided in Supplementary Table 2.

#### DICOM to BIDS tag converter

This configuration file provides the DICOM tag name for each required BIDS tag compensating for variable naming conventions between the two formats. If no DICOM tag equivalent exists for a given BIDS requirement, the sequence overview or PET-doses replacement option is triggered. Description and examples of the configuration file are given in Supplementary Table 3.

### Data quality assessment

To provide an indication of the quality of the database formatted to BIDS standards, we analyzed the distribution of three relevant metrics: SNR and CNR for MRI acquisitions, and median intensity for PET and MRI acquisitions. The metrics were measured on voxels with tissue signal (volume of interest, VOI) following Otsu thresholding on the volume with the highest intensity^28^. Voxels below the given threshold were considered background for noise measurement. The metrics were calculated using the following formulas:

$${{\mathrm{SNR}}}=\frac{{\mu }_{{{\mathrm{VOI}}}}}{{\sigma }_{{{\mathrm{noise}}}}}$$

$${{\mathrm{CNR}}}=\frac{{{\mathrm{ma}}}{{\mathrm{x}}}_{{{\mathrm{VOI}}}}-{{\mathrm{mi}}}{{\mathrm{n}}}_{{{\mathrm{VOI}}}}}{{\sigma }_{{{\mathrm{noise}}}}}$$

$${{\mathrm{Median}}}\, {\mathrm{intensity}}={{\mathrm{media}}}{{\mathrm{n}}}_{{{\mathrm{VOI}}}}$$

Categorical violin plots were plotted for each metric. These plots show the distribution of the metric values across subjects. Strip plots were superimposed on the violin plots to provide additional information on time distributions. In these plots, each category corresponds to an MRI sequence or PET tracer, and the points represent the metric values for each acquisition within that sequence. For dynamic MRI sequences and PET acquisitions, we averaged the metrics across time points or frames and plotted the resulting values. For multi-echo sequences, we averaged the median of intensity across echo times and plotted the resulting values. In terms of noise evaluation, only the first echo was plotted as the SNR is maximal at this echo time. Additionally, we provided a specific PET metric based on counting statistics before reconstruction, enabling the calculation of the PNECR (PNECR = (*P* − *D*)^2^/*P*, with *P* total prompts and *D* total randoms)^29^. The maximum of PNECR was represented following the representation methods of the previously described metrics. The dynamic evolution of PNECR over time was represented on a logarithmic scale to uncover variations in tracer injection times. Lastly, a sample of the data was manually inspected to ensure that the results were accurate and to identify any issues that might have been missed. Based on these evaluations, we were able to provide an indication of the overall quality of the database for potential future users of the dataset, including the detection of potential outliers and the assessment of the homogeneity and consistency of the data.

### Execution protocol

Detailed protocol steps to execute the formatting script are provided on protocol.io^30^.

### Reporting summary

Further information on research design is available in the Nature Portfolio Reporting Summary linked to this article.

## Online content

Any methods, additional references, Nature Portfolio reporting summaries, source data, extended data, supplementary information, acknowledgements, peer review information; details of author contributions and competing interests; and statements of data and code availability are available at 10.1038/s41684-023-01289-9.

### Supplementary information

Supplementary Information Supplementary Fig. 1 and Tables 1–3.

Reporting Summary

## Data Availability

The dataset supporting the results of this article is available in the PRIME-DE repository, with identifier ‘pending identifier’ and licensed under Data Usage Agreement (https://fcon_1000.projects.nitrc.org/indi/PRIME/carmen-lyon.html).

## Code availability

The code is hosted in a Public GitLab repository under the name cermep-bids-retro (https://gitlab.in2p3.fr/cermep/cermep-bids-retro). It is fully implemented in the Python programming language and is operating system independent. The Python environment requirements are provided in the environment.yml file. The code is licensed under MIT.

## References

[1] Gorgolewski KJ (2016). The brain imaging data structure, a format for organizing and describing outputs of neuroimaging experiments. Sci. Data.

[2] Wilkinson MD (2016). The FAIR Guiding Principles for scientific data management and stewardship. Sci. Data.

[3] Poldrack RA (2017). Scanning the horizon: towards transparent and reproducible neuroimaging research. Nat. Rev. Neurosci..

[4] Friedrich P (2021). Imaging evolution of the primate brain: the next frontier?. NeuroImage.

[5] Phillips KA (2014). Why primate models matter. Am. J. Primatol..

[6] Aske KC, Waugh CA (2017). Expanding the 3R principles. EMBO Rep..

[7] Milham M (2020). Accelerating the evolution of nonhuman primate neuroimaging. Neuron.

[8] Subbaraman N (2021). The US is boosting funding for research monkeys in the wake of COVID. Nature.

[9] O’Grady C (2022). Airline’s decision to end monkey transports will worsen shortage in research. Science.

[10] Milham MP (2018). An open resource for non-human primate imaging. Neuron.

[11] Knudsen GM (2020). Guidelines for the content and format of PET brain data in publications and archives: a consensus paper. J. Cereb. Blood Flow Metab..

[12] Norgaard M (2022). PET-BIDS, an extension to the brain imaging data structure for positron emission tomography. Sci. Data.

[13] Mérida I (2021). CERMEP-IDB-MRXFDG: a database of 37 normal adult human brain [^18^F]FDG PET, T1 and FLAIR MRI, and CT images available for research. EJNMMI Res..

[14] Wintermark M (2005). Comparative overview of brain perfusion imaging techniques. Stroke.

[15] Chauveau F, Becker G, Boutin H (2021). Have (R)-[^11^C]PK11195 challengers fulfilled the promise? A scoping review of clinical TSPO PET studies. Eur. J. Nucl. Med. Mol. Imaging.

[16] Becker G (2023). Spatio-temporal characterization of brain inflammation in a non-human primate stroke model mimicking endovascular thrombectomy. Neurotherapeutics.

[17] Autio JA (2021). Minimal specifications for non-human primate MRI: challenges in standardizing and harmonizing data collection. NeuroImage.

[18] Debatisse J (2021). A non-human primate model of stroke reproducing endovascular thrombectomy and allowing long-term imaging and neurological read-outs. J. Cereb. Blood Flow Metab..

[19] Debatisse J (2020). PET-MRI nanoparticles imaging of blood–brain barrier damage and modulation after stroke reperfusion. Brain Commun..

[20] Kilkenny, C., Browne, W. J., Cuthill, I. C., Emerson, M. & Altman, D. G. Improving bioscience research reporting: the ARRIVE guidelines for reporting animal research. *PLoS Biol.* **8**, e1000412 (2010). 10.1371/journal.pbio.1000412 PMC2893951 20613859

[21] Percie du Sert N (2020). The ARRIVE guidelines 2.0: updated guidelines for reporting animal research. PLoS Biol..

[22] Bedetti, C. et al. UNFmontreal/Dcm2Bids: 2.1.8. *Zenodo*10.5281/zenodo.6658099 (2022)

[23] Mason, D. et al. pydicom/pydicom: pydicom 2.3.1. *Zenodo*10.5281/zenodo.7319790 (2022)

[24] Brett, M. et al. nipy/nibabel:. *Zenodo*10.5281/zenodo.6658382 (2022).

[25] Yushkevich PA, Gao Y, Gerig G (2016). ITK-SNAP: an interactive tool for semi-automatic segmentation of multi-modality biomedical images. Annu. Int. Conf. IEEE Eng. Med. Biol. Soc..

[26] icometrix/dicom2nifti. *GitHub* https://github.com/icometrix/dicom2nifti (2022).

[27] Luurtsema G (2021). EANM guideline for harmonisation on molar activity or specific activity of radiopharmaceuticals: impact on safety and imaging quality. EJNMMI Radiopharm. Chem..

[28] Otsu N (1979). A threshold selection method from Gray-level histograms. IEEE Trans. Syst. Man Cybern..

[29] Watson CC (2003). Evaluation of clinical PET count rate performance. IEEE Trans. Nucl. Sci..

[30] cermep-bids-retro V.1. *protocols.io* https://www.protocols.io/view/cermep-bids-retro-261ge319dl47/v1 (2023).

