# Supplementary material for: The PREMISE database of 20 Macaca fascicularis PET/MRI brain images available for research
Source: Lab Anim (NY). 2023 Nov 23;53(1):13–7. doi: 10.1038/s41684-023-01289-9 (PMC10766538; doi:10.1038/s41684-023-01289-9)
Supplement: Supplementary file 1 — Supplementary Fig. 1 and Tables 1–3. [file 41684_2023_1289_MOESM1_ESM.pdf]

nature portfolio

https://doi.org/10.1038/s41684-023-01289-9

Supplementary information

The PREMISE database of 20 *Macaca fascicularis* PET/MRI brain images available for research

In the format provided by the authors and unedited

The PREMISE database of 20 *Macaca Fascicularis* PET/MRI brain images available for research

Chalet et al.

Supplementary data

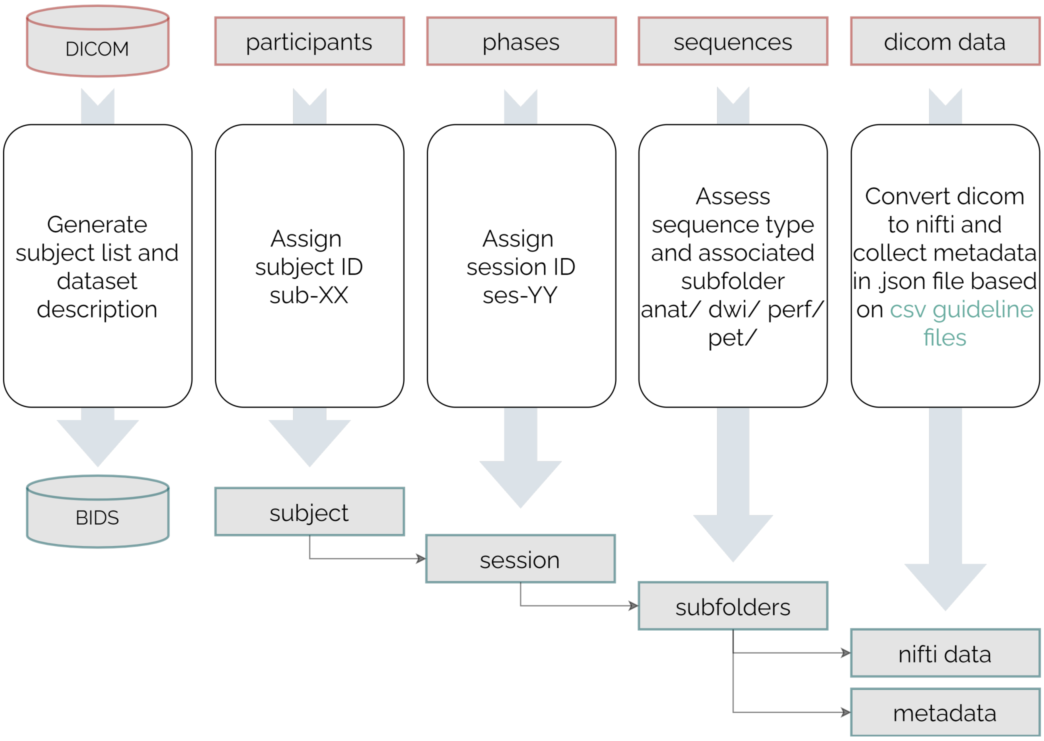

Figure 1. Processing pipeline tasks overview to convert raw DICOM structure to BIDS structure. This pipeline was implemented in python and can be run through a single command line taking DICOM database path input along with parameter files. The input structure of the dataset may vary as data can be grouped by subjects, acquisition type or protocol phase in any given order.

Compiled on: October 10, 2023.

Table 1. Sequence overview configuration file description and examples.

sequences	src	filename	folder	trc	bids-tag1 PulseSequence	bids-tag2 EchoTime	bids-tagX InjectedMass
Sequence name in BIDS standards	Variable name of location folder for database in DICOM format	Sequence name in DICOM database	Storing folder in BIDS standards	Tracer name for PET data	Example of BIDS required metadata for each sequence. These tags will be integrated in the JSON file upon formatting following instructions: 0- non-required tag; 1- required tag available in DICOM file; 2- required tag available with additional formatting steps; str- replacement value for missing tag; file- subject specific value available in PET configuration file.		
T2w<br>trc-PK11195	raw_mri<br>raw_pet	SE_32TE<br>PK11195	anat<br>pet	0<br>PK11195	Spin Echo<br>0	2<br>0	0<br>file

1st row: header; 2nd row: column description; 3rd row: examples for MRI and PET acquisitions.
A value is attributed to each tag depending on the sequence-specific requirements, the tags availability and format in the DICOM metadata and the modality:

- 0- Tag not included in the JSON metadata file of the sequence.
- 1- Tag included in the JSON metadata file of the sequence as an exact copy of the equivalent DICOM tag.
- 2- Tag included in the JSON metadata file of the sequence as a formatted copy of the equivalent DICOM tag.
- str- Tag value manually set for the JSON metadata file due to a missing value in the DICOM file.
- "file"- Variable tag throughout subjects whose value must be extracted from the "PET doses" file.

Table 2. PET doses configuration file description and examples.

subject	session	trc	Specific Radioactivity	Injected Radioactivity	InjectedMass
Subject name in DICOM database	Session name in DICOM database	Tracer name as set in sequence overview configuration file	Measured specific radioactivity in units specified in BIDS tag SpecificRadioactivityUnits	Measured injected radioactivity in units specified in BIDS tag InjectedRadioactivityUnits	Calculated injected mass in units specified in BIDS tag InjectedMassUnits
SUBJECT-XA<br>SUBJECT-XB<br>SUBJECT-XB	Baseline<br>Baseline<br>Post-op	H2O15<br>PK11195<br>PK11195	na<br>49.8<br>48.9	259<br>138<br>126	na<br>2.77<br>2.58

1st row: header; 2nd row: column description; 3rd row: examples for PET tracers and sessions.

Table 3. DICOM to BIDS tags conversion configuration file description and examples.

bids-tag	dicom-tag
BIDS tag naming convention	DICOM tag equivalent
Manufacturer<br>TimeZero<br>PulseSequence	Manufacturer<br>AcquisitionTime<br>None*

1st row: header; 2nd row: column description; 3rd row: example of tags
*tag not in DICOM metadata: refer to sequence overview configuration file
